# Carbon monoxide affects electrical and contractile activity of rat myocardium

**DOI:** 10.1186/1423-0127-18-40

**Published:** 2011-06-15

**Authors:** Denis V Abramochkin, Nail N Haertdinov, Maria V Porokhnya, Andrew L Zefirov, Gusel F Sitdikova

**Affiliations:** 1 Department of Human and Animal Physiology, Moscow State University, Moscow, Russia; 2 Kazan Federal University, Kazan, Russia; 3 Kazan State Medical University, Kazan, Russia

## Abstract

**Background:**

Carbon monoxide (CO) is a toxic gas, which also acts in the organism as a neurotransmitter. It is generated as a by-product of heme breakdown catalyzed by heme oxygenase. We have investigated changes in electrical and contractile activity of isolated rat atrial and ventricular myocardium preparations under the influence of CO.

**Methods:**

Standard microelectrode technique was used for intracellular registration of electrical activity in isolated preparations of atrial and ventricular myocardium. Contractions of atrial myocardial stripes were registered via force transducer.

**Results:**

CO (10^-4 ^- 10^-3 ^M) caused prominent decrease of action potential duration (APD) in working atrial myocardium as well as significant acceleration of sinus rhythm. In addition CO reduced force of contractions and other parameters of contractile activity. Inhibitor of heme oxygenase zinc protoporphyrin IX exerts opposite effects: prolongation of action potential, reduction of sinus rhythm rate and enhancement of contractile function. Therefore, endogenous CO, which may be generated in the heart due to the presence of active heme oxygenase, is likely to exert the same effects as exogenous CO applied to the perfusing medium. In ventricular myocardium preparations exogenous CO also induced shortening of action potential, while zinc protoporphyrin IX produced the opposite effect.

**Conclusions:**

Thus, endogenous or exogenous carbon monoxide may act as an important regulator of electrical and contractile cardiac activity.

## Background

During the last twenty years two toxic gases: carbon monoxide (CO) and hydrogen sulfide (H_2_S) were recognized as important regulatory compounds which are synthesized endogenously in different tissues of the organism [1]. Along with nitric oxide (NO) CO and H_2_S represent a class of gaseous transmitters. Cardiovascular effects of NO, CO and H_2_S have been extensively studied. For instance, all three gases act as potent vasodilators though NO and CO relax vascular smooth muscle cells in a few orders of magnitude lower concentrations than H_2_S [1]. The effect of gaseous mediators on heart functioning has received less attention than their effect on blood vessels. NO donors produce positive inotropic effect in low concentrations, while higher concentrations may cause suppression of contractile activity [2]. These compounds also accelerate the sinoatrial node rhythm and therefore increase the heart rate [1]. Such effects of NO are mediated by modulation of numerous ionic currents (I_Ca_, I_Ks_, I_K1_, I_f _and others) via either activation of soluble guanylyl cyclase with subsequent increase of intracellular cGMP content and cGMP-independent mechanisms (S-nitrosylation and direct effects on G proteins) [3]. In contrast to NO, H_2_S exhibits negative inotropic activity in all examined concentrations [4], it also reduces action potential duration (APD) in rat atrial myocardium [5]. Cardioprotective effects of all three gases against ischemic injury have been described in several studies [6-8].

Cardiotropic effects of CO have been sparsely investigated and the data are quite controversial. While some authors report negative inotropic effect of CO [9], others describe positive inotropy in isolated rat heart [7]. Nothing is known about possible CO-induced changes of cardiac electrical activity. Nevertheless, investigation of cardiotropic effects of CO is interesting especially in the light of cardioprotective action of CO against ischemia-reperfusion injury shown in several studies [10,11]. Therefore, the aim of the present study was to explore effects of exogenous and endogenous CO on the electrical and contractile activity of isolated rat myocardium preparation.

## Methods

The investigation conformed with the *Guide for the Care and Use of Laboratory Animals *published by the US National Institutes of Health (NIH publication no. 85-23, revised 1996), and the experimental protocol was approved by the Bioethics Committee of Moscow State University and the Animal Care and Use Committee of Kazan State Medical University.

Male Wistar rats (*n *= 38) weighing 280-320 g were decapitated using a guillotine for small animals (OpenScience, Moscow, Russia), the chest was opened and the heart rapidly excised and immersed in a physiological solution containing (mM): NaCl, 130.0; KCl, 5.6; NaH_2_PO_4_, 0.6; MgCl_2_, 1.1; CaCl_2_, 1.8; NaHCO_3_, 20.0 and glucose, 11.0, bubbled with carbogen (95% O_2_-5% CO_2_), with pH 7.4 ± 0.1.

### Intracellular recordings of APs in isolated right atrium and right ventricular wall of rats

The right atrial preparation, including the auricle, the crista terminalis, the intercaval region and the sinoatrial node, was isolated and pinned to the bottom of an experimental chamber (3 ml) supplied with a physiological solution at 10 ml min^-1 ^(37.5°C). After 2 h of equilibration, transmembrane potentials were recorded with glass microelectrodes (20-30 MΩ) filled with 3 M KCl. The signal was digitized and analyzed using specific software (DISoft, Moscow, Russia; Synaptosoft, Decatur, GA, USA). Spontaneously occurring action potentials (APs) were recorded from the endocardial surface of the auricle. In some experiments the sinoatrial node was removed and the preparation was paced (6 Hz) via silver teflon-coated electrodes. Stable impalements were maintained during the entire period action of the drugs. Changes in the cycle length (CL) and the AP duration to 50 (APD50) and 90% of repolarization (APD90) were analyzed. The similar experiments were performed using isolated right ventricular wall preparations paced with a frequency of 6 Hz. APs were also recorded from subendocardial layers.

### Registration of contractile activity

The stripes of working myocardium of 4-6 mm length and 1 mm diameter were excised from the right atrium preparations and mounted in a special perfusion chamber (ADInstruments, Sydney, Australia). During the experiment it was stimulated with a frequency of 0.1 Hz via pair of silver electrodes. The force of contractions was registered using a mechanotransducer MLT 050/D (ADInstruments, Sydney, Australia). The data were analyzed with Chart v.5.0 (ADInstruments, Sydney, Australia) software. The amplitude of contractions was determined as well as maximal velocity of contraction (MVC) and relaxation (MVR).

### Materials

CO of 99% purity was obtained from NIIKM (Moscow, Russia). The inhibitor of heme oxygenase (HO) zinc protoporphyrin-IX (ZnPP) was purchased from Sigma (St Louis, MO, USA).

The stock solution of CO was prepared immediately before its usage in the experiment by bubbling of physiological solution with CO. Since the water solubility of CO is 26.91 mg/l, the concentration of CO in the stock solution was 0.96 mM [12]. To simplify the presentation of data we considered it to be 1 mM approximately. During the experiment the stock solution was used to prepare physiological solutions with different concentrations of CO which were applied to the experimental chamber. The stock solution of ZnPP was prepared as described by Vreman et al [13]. Experiments with ZnPP were carried out at a very low or zero ambient light due to the photo reactivity of compound [12].

### Data analysis

All results in the text, tables and figures are expressed as means ± S.E.M. for *n *experiments. All samples were tested with Kolmogorov-Smirnov normality test. Each sample differed significantly from the normal distribution (*P <*0.05 for every sample), so we used non-parametric tests for analysis. The effects of CO or ZnPP on electrophysiological and mechanical parameters were compared with respective basal values of these parameters by Wilcoxon signed rank test.

## Results

### Effects of exogenous CO on electrical activity of atrial and ventricular myocardium

**Figure 1:**
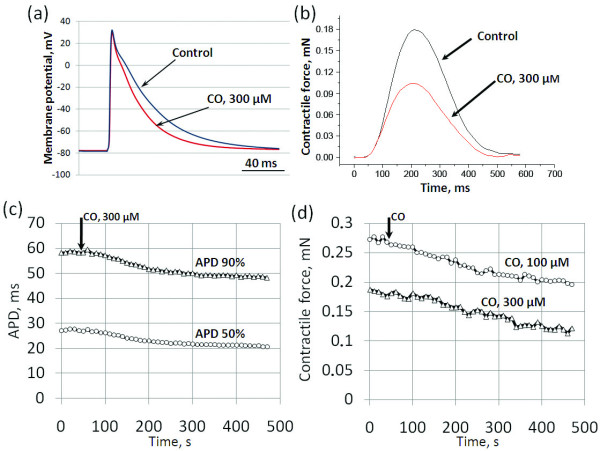
**Changes in configuration of electrical and contractile activity of atrial myocardium induced by CO**. Representative original traces of (a) action potentials (APs) in spontaneously beating right atrial preparations and (b) contraction curves in paced (0.1 Hz) stripes of working right atrial myocardium during control conditions and 300 µM CO. Relationship between decrease in (c) AP duration (APD) and (d) contractile force and the time of CO application-data from separate representative experiments. The black arrow indicates the moment of CO application.

**Figure 2:**
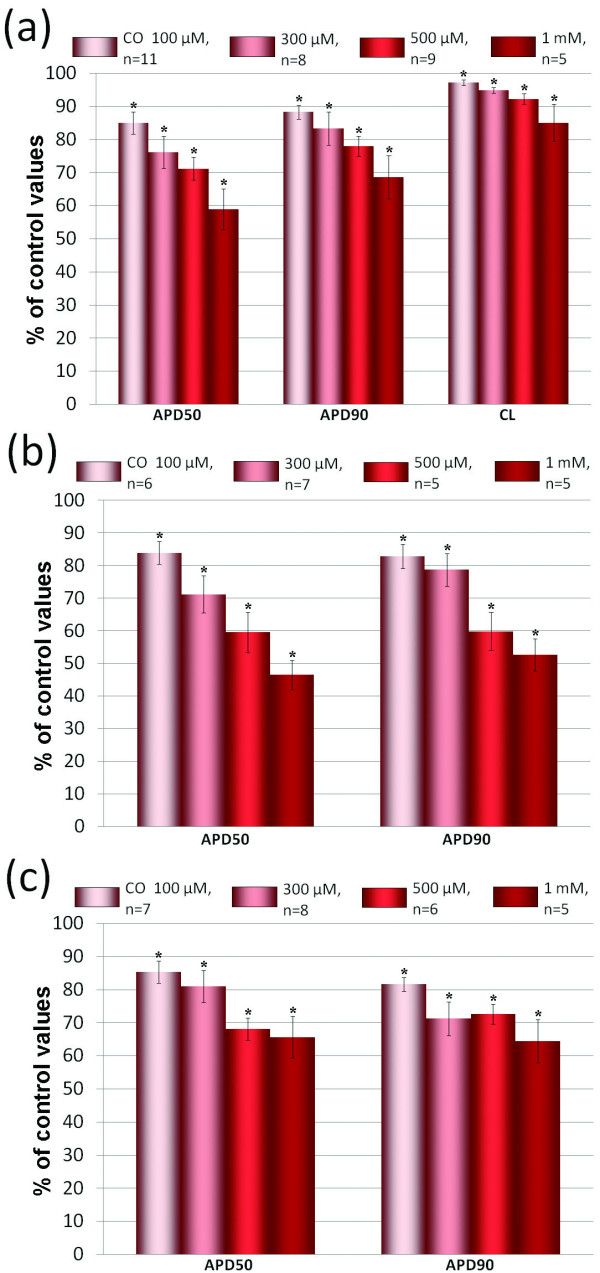
**Alteration of electrophysiological parameters by CO**. Effects of CO (10^-4 ^- 10^-3^M) on APD and cycle length (CL) in (a) spontaneously beating atrial preparations, (b) paced atrial preparations and (c) paced ventricular preparations. The control values of APD50, APD90 and CL were considered as 100%. The relative values of these parameters during action of CO are presented in % of control. * - p < 0.05 *versus *the respective control values.

In the initial stage of our study, we investigated the modulation of APD and CL in spontaneously beating right atrium preparations during 8 min superfusion by solution containing CO in different concentrations. During the control conditions, APD50, APD90 and CL were 28.7 ± 3.6, 58.2 ± 5.3 and 205 ± 18.7 ms, respectively. 5 × 10^-5 ^M CO didn't alter any of these parameters significantly. However, the application of higher CO concentrations (10^-4^, 3 × 10^-4^, 5 × 10^-4 ^and 10^-3^M) produced a marked decrease in APD50 and APD90 (Figures 1a, b and 2a) as well as in CL (Figure 2a). These effects of CO were rapidly developing, reaching the maximum after 5 min of superfusion (Figure 1c). The time course of development of the effect was similar in all subsequent electrophysiological experiments with CO, so we discuss only the maximal values of AP shortening and reduction of the CL. No significant changes of the resting membrane potential were registered in any of the electrophysiological experiments with CO or ZnPP.

It is well known that increase of the beating rate *per se *leads to the reduction of APD. Therefore, shortening of AP under the action of CO in spontaneously beating preparations may be due to increase of sinoatrial node rate. To check this possibility we have performed a series of experiments with paced atrial preparations. In these experiments CO reduced APD50 and APD90 as much as in spontaneously beating preparations (Figure 2b).

In working myocardium of isolated right ventricular wall CO also induced marked shortening of APs (Figure 2c).

### Effects of CO on contractile activity of atrial myocardium

**Figure 3:**
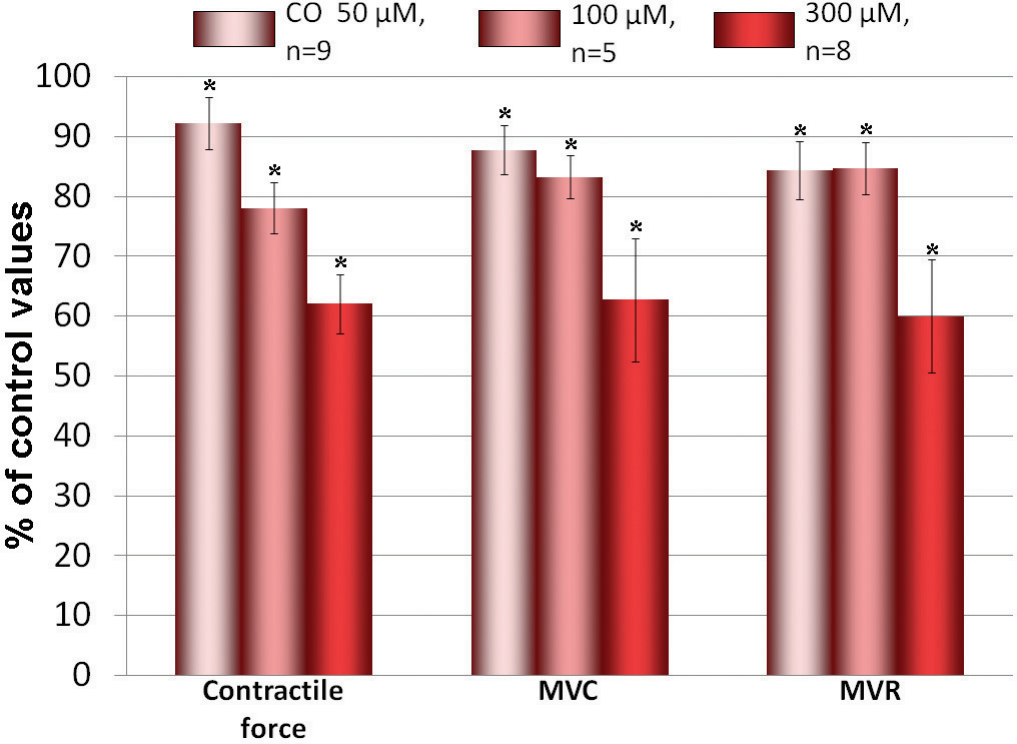
**Alteration of contractile parameters by CO**. Effects of CO (5 × 10^-5 ^- 3 × 10^-4^M) on contractile force, maximal velocity of contraction (MVC) and maximal velocity of relaxation (MVR) in paced stripes of working atrial myocardium. For all columns p < 0.05 *versus *the respective control values.

It is generally accepted that modulation of electrical activity may cause substantial changes in force of contractions. Therefore, we studied effects of CO on parameters of contractions of isolated right atrial myocardium stripes. All tested concentrations of CO (5 × 10^-5^, 10^-4 ^and 3 × 10^-4^M) produced dose-dependent reduction of three most informative parameters of contractile activity: amplitude of contractions, MVC and MVR (Figure 1b,d, 3). Thus, exogenous CO suppresses contractile activity of atrial myocardium in concentrations close to those, which are effective in point of electrophysiological parameters.

### Effects of HO inhibitor ZnPP

**Figure 4:**
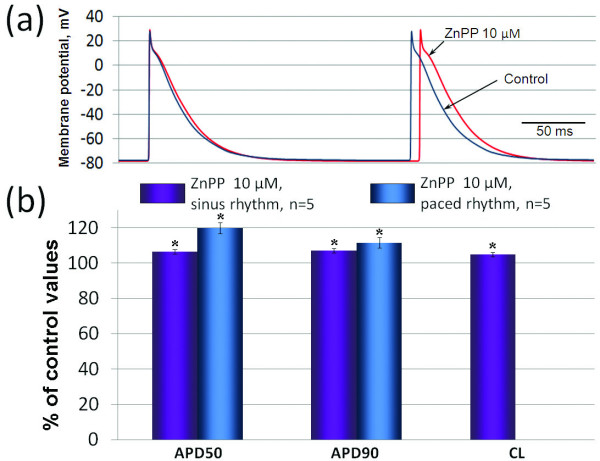
**Changes in electrical activity induced by zinc protoporphyrin IX**. Effects of CO (5 × 10^-5 ^- 3 × 10^-4^M) on contractile force, maximal velocity of contraction (MVC) and maximal velocity of relaxation (MVR) in paced stripes of working atrial myocardium. The control values of contractile force, MVC and MVR were considered as 100%. The relative values of these parameters during action of CO are presented in % of control. * - p < 0.05 *versus *the respective control values.

**Figure 5:**
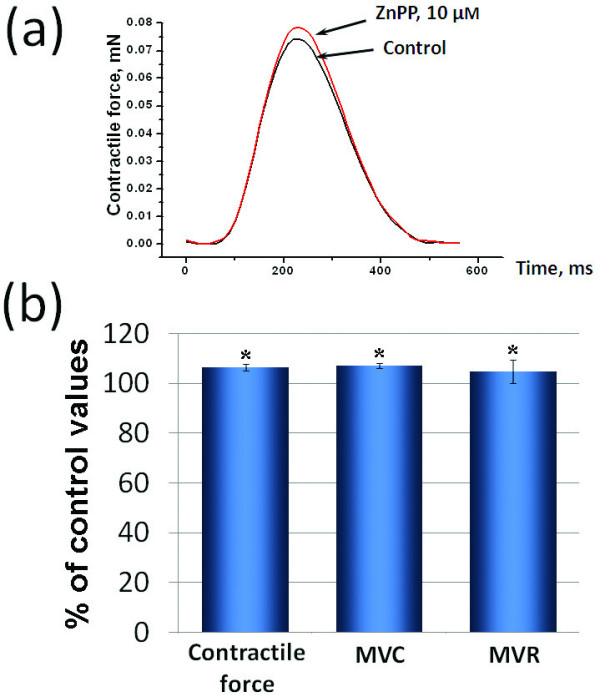
**Changes in contractile activity induced by zinc protoporphyrin IX**. Effects of ZnPP on contractile activity of working atrial myocardium: (a) representative traces of contraction curves during control conditions and 10 µM ZnPP, (b) maximal effects of ZnPP on contractile force MVC and MVR (n = 5). In part (b) the control values of contractile force, MVC and MVR were considered as 100%. The relative values of these parameters during action of ZnPP are presented in % of control. * - p < 0.05 *versus *the respective control values.

We have tried to determine the role of CO endogenously produced in the myocardium by blocking its synthesis using application of 10^-5 ^M ZnPP, inhibitor of HO. This dose was based on previous studies demonstrating reliable HO activity inhibition at this concentration [13,14]. ZnPP produced several effects that were all opposite to the effects of exogenous CO. In spontaneously beating atrial preparations it caused increase in APD50 and APD90 and prolongation of CL (Figure 4). These effects of 10^-5 ^M ZnPP were significant, but not as prominent as in the case of exogenous CO. In paced atrial (Figure 4b) and ventricular (data not shown) preparations ZnPP also induced prolongation of AP repolarization. In experiments with registration of mechanical activity we observed significant increase in amplitude of contractions, MVC and MVR (Figure 5).

## Discussion

In the present study we provide the first to our knowledge evidence that CO significantly affects electrical activity of atrial and ventricular myocardium. We demonstrate the striking difference between action of CO in the working myocardium, where it produces negative effects: shortening of APs and suppression of contractile activity, and sinoatrial node. Unlike H_2_S [5], CO accelerates the sinoatrial rhythm. It seems that signal transduction pathways, which are activated by CO, may differ in working and sinoatrial cardiomyocytes. For example, variations in enzymes, which synthesize and degrade second messengers like cAMP and cGMP, may be present in these types of cardiac cells. Further investigation of signal transduction pathways mediating cardiotropic effects of CO will help to reveal the mechanisms of such opposite effects of CO in working and pacemaker myocardium.

The described negative inotropic effect of CO conforms to the findings of Liu et al that demonstrated suppression of rat papillary muscles contractions under 1 mM CO [9]. However, our data are in controversy with results of Musameh et al showing the positive inotropy of CO associated with decrease of heart rate in the isolated rat Langendorf-perfused heart preparation [7]. The latter discrepancy might be at least partly due to the difference in the method of CO application to the perfusing solution. Like Liu et al we used the stock solution directly saturated with CO, while Musameh et al used special CO releasing molecules that may possibly exert some accessory effects. Mechanisms of both negative and positive effects of CO need special investigation. Negative effects on APD and contractile parameters may be partly explained by suppression of I_CaL _by CO [15], although this assumption should be tested further. Unfortunately, blockers of calcium channels drastically modify electric activity of myocardium. For example, nifedepine produces extremely large shortening of APs and slowing of sinus rhythm if used in concentration sufficient for full block of I_CaL_. Therefore, effects of these compounds will mask possible action of CO. So, we guess that the only way to prove this assumption is to perform appropriate patch-clamp experiments.

Although exogenous CO causes prominent changes in the electrical and mechanical activity of myocardium, it doesn't testify to the physiological role of CO as a regulator of cardiac performance. To act as a cardioregulator CO should be synthesized in the myocardium or in the close proximity to it, because it is quickly scavenged in the organism by hemoglobin and other heme proteins [16]. CO is generated in the organism as a by-product of heme breakdown catalyzed by HOs: inducible HO-1 and constitutively expressed HO-2. In normal conditions expression of HO-1 is detected in heart vascular wall, but not in cardiomyocytes [16]. However, HO-1 protein expression may be significantly up-regulated by pathological stimuli, such as myocardial infarction [17], or hypoxia [18]. HO-2 is expressed in the atrial and ventricular myocardium [19] as well as in intracardiac neurons [20]. Thus, heart possesses potential sources of endogenous CO. Experiments with ZnPP, which inhibits both isoforms of HO, clearly indicate that inhibition of endogenous CO production leads to significant changes in electrical and contractile activity of myocardium which are opposite to the effects of exogenous CO. Therefore, endogenous CO seems to be synthesized in the myocardium in normal conditions and act similarly to the exogenous CO, although it is still not clear if cardiac CO is synthesized in the cardiomyocytes by HO-2 or in the coronary vessels by HO-1.

Unfortunately, the present study has several limitations. First, the concentrations of CO sufficient for the significant alteration of cardiac electrical and mechanical activity in our experiments are strikingly high. CO is transported by blood in the form of carbonmonoxy-hemoglobin A (COHb). Estimated physiological concentrations of CO in tissues are rather low, in the nanomolar range if based on normal levels of COHb of 1 to 2% [21]. However, the correlation between COHb and tissue concentrations of CO is questionable, since CO generated in living cells would first be scavenged in the cytosol by heme proteins reaching the bloodstream [16]. In fact, local concentrations of CO might be as high as concentrations used in our experiments. The significant effects of ZnPP validate this assumption, although the possibility of non-specific ZnPP action can't be excluded. However, while high concentrations of ZnPP (10^-4 ^M) are really able to inhibit guanylyl cyclase [22] and calcium channels [23], 10^-5 ^M ZnPP is considered as a quite selective HO inhibitor [22]. Moreover, inhibition of L-type calcium current leads to shortening of APs, while ZnPP induces increase in APD.

## Conclusions

Thus, we have shown that both exogenous and endogenous CO substantially alters electrical and contractile activity of rat heart by decreasing the APD in working atrial and ventricular myocardium, increasing the sinoatrial node beating rate and suppressing the contractile activity of atrial myocardium.

## Competing interests

The authors declare that they have no competing interests.

## Authors' contributions

DVA carried out the electrophysiological experiments and prepared the manuscript. NNH carried out experiments with registration of contractile activity. MVP participated in the electrophysiological experiments. ALZ participated in the design of the study. GFS participated in the design of the study and preparation of the manuscript. All authors read and approved the final manuscript.
